# The State Dental Association of Kansas

**Published:** 1873-06

**Authors:** A. H. Thompson


### THE STATE DENTAL ASSOCIATION OF KANSAS.

The second annual convention of the State Dental Association was held at Laurence, on the 6th, 7th, and 8th of May.

The Association met at 2 P. M., May 6th, in the parlors of the Elridge House, and was called to order by J. B. Wheeler, President of the Association.

Dr. L. C. Wasson, of Ottawa ; C. H. Reeder, of Burlingame ; Geo. C. Holmes, of Laurencc, and H. T. Wilson, of Parsons, were elected to active membership.

The following officers were elected for the ensuing year :

President—Dr. W. F. Griswold, of Leavenworth ; Vice President—Dr. J. D. Patterson, of Laurence ; Recording Secretary—A. H. Tompson, of Topeka ; Corresponding Secretary—L. C. Wasson, of Ottawa, Treasurer ;—A. Doud, of Olathe.

The following Standing Committees were appointed for the ensuing year.

Executive Committee—Drs. A. M. Callaghan, C. H. Rudder and Geo. C. Holmes ; Committee on Membership—Drs. G. H. Dubois, J. R. Boyd and A. Doud ; Operative Dentistry—J. B. Wheeler, J. D. Patterson and L. C. Wasson ; Mechanical Dentistry—Drs. A. H. Thompson, A. C. McNary and S. O. Freman ; Publication and Literature—A. H. Thompson, J. B Wheeler, J. D. Patterson and L. C. Wasson.

Drs. T. F. Griswold and J. B. Wheeler were elected delegates to the American Dental Association.

After which the Society listened to the reading of several very interesting essays, which were followed by discussions in which nearly all took a pait.

After spending three days very profitably to all we hope, the Association adjourned to meet at Ottawa, on the first Tuesday in October next.

A. H. Thompson,
Recording Secretary.

